# The Inhibition of NS2B/NS3 Protease: A New Therapeutic Opportunity to Treat Dengue and Zika Virus Infection

**DOI:** 10.3390/ijms25084376

**Published:** 2024-04-16

**Authors:** Josè Starvaggi, Santo Previti, Maria Zappalà, Roberta Ettari

**Affiliations:** Department of Chemical, Biological, Pharmaceutical, and Environmental Sciences, University of Messina, Viale Ferdinando Stagno d’Alcontres 31, 98166 Messina, Italy; starvaggi4@gmail.com (J.S.); spreviti@unime.it (S.P.); mzappala@unime.it (M.Z.)

**Keywords:** dengue virus, zika virus, NS2B/NS3 serine protease, antiviral agents, orthosteric and allosteric inhibitors

## Abstract

In the global pandemic scenario, dengue and zika viruses (DENV and ZIKV, respectively), both mosquito-borne members of the flaviviridae family, represent a serious health problem, and considering the absence of specific antiviral drugs and available vaccines, there is a dire need to identify new targets to treat these types of viral infections. Within this drug discovery process, the protease NS2B/NS3 is considered the primary target for the development of novel anti-flavivirus drugs. The NS2B/NS3 is a serine protease that has a dual function both in the viral replication process and in the elusion of the innate immunity. To date, two main classes of NS2B/NS3 of DENV and ZIKV protease inhibitors have been discovered: those that bind to the orthosteric site and those that act at the allosteric site. Therefore, this perspective article aims to discuss the main features of the use of the most potent NS2B/NS3 inhibitors and their impact at the social level.

## 1. Introduction

In the global pandemic scenario, dengue virus (DENV) diffusion has become a serious health problem in the current years with an estimated number of cases of about 100–400 million annually [1]. Dengue is endemic in more than 100 countries belonging to Africa, America, Eastern Mediterranean, South-East Asia, and Western Pacific; it is classified among the Neglected Tropical Diseases (NTDs) [2].

DENV infection, also known as “bone-break fever”, could be characterized by a broad spectrum of clinical symptoms, including uncomplicated fever (dengue fever), that normally begin 4–10 days after infection, such as headache, lethargy, muscle and joint pains, nausea, vomiting, confusion or, in a few cases, more serious clinical complications like dengue hemorrhagic fever (DHF) and dengue shock syndrome (DSS), which are potentially fatal for the patient [3].

Zika virus (ZIKV) infection, not officially categorized as an NTD, was firstly identified in Uganda in 1947, and since 2007, outbreaks of ZIKV disease were recorded in Africa, America, and Asia [4]. The ZIKV infection typically develops common symptoms 2–7 days after infection, like fever, headache, muscle pain, and conjunctivitis [5]. No hemorrhagic events were associated with ZIKV fever that, as discussed before, are typical of dengue. The most serious complication of this infection is the Guillain-Barrè syndrome (GBS), a demyelinating disease of the peripheral nervous system, able to induce devastating paralysis [5]. An infection during pregnancy can lead to early miscarriages, intrauterine fetal demise, impaired fetal growth, and placental dysfunction. Moreover, in kids born from ZIKV-infected mothers during pregnancy, it was possible to observe congenital zika syndrome (CZS): microcephaly and other congenital malformations in the infant, including limb contractures, eye abnormalities, and hearing loss [5]. 

Both viruses are transmitted by the mosquitoes *Aedes albopictus* and *Aedes aegypti* that recently are prevalent also in the temperate climate zone, starting from 2010 leading to dengue transmission in continental Europe, such as France and Croatia, with 1043 cases of DENV infection diagnosed in Italy from 2010–2021. Currently, there are no vaccines or antiviral drugs available for these viral infections. 

## 2. NS2B/NS3 DENV and ZIKV Protease: Structures and Functions

DENV is a mosquito-borne flavivirus infection, mainly transmitted by *Aedes aegypti* and *Aedes albopictus* mosquitoes, and after the mosquito bite, the virus lays down on skin epidermis encountering keratinocytes and Langerhans cells, which are highly permissive to virus entrance [5].

There are four serotypes of DENV, which are antigenically classified as DENV-1, DENV-2, DENV-3, and DENV-4 [3]. DENV belongs to the flaviviridae family, it is an RNA virus, and its genome is formed by a positive-sense single-stranded RNA (+ssRNA), with a size of about 11 Kb [2]. The viral genome encodes for three structural proteins, the precursor membrane (prM), the envelope proteins (E), and the capsid (C), and seven non-structural proteins (NS1, NS2A, NS2B, NS3, NS4A, NS4B, and NS5). The whole genomic RNA is translated into a single large precursor polyprotein that is cleaved by the viral NS2B/NS3 serine protease and by host proteases into functional proteins. 

The three structural proteins, i.e., prM, E, and C, are crucial to form the viral particles: the C protein takes part in the composition of the viral icosahedral capsid, while the M and E proteins form transmembrane helices that help the viral particles to anchor on the membrane surface. The E protein is the principal viral protein involved in host cell membrane fusion during the interaction with the host receptor [5]. The seven non-structural proteins, essential for viral replication and maturation, are the viral protease (NS2B/NS3), the helicase (NS3), the methyltransferase (NS5), the RNA-dependent RNA polymerase (NS5), NS1 and NS2 that are involved in the viral replication, while NS4 is involved in the membrane alteration [6]. The most important non-structural protein involved in the pathogenesis of dengue viral infections is NS1 [3].

The NS2B/NS3 is a trypsin-like serine protease showing a dual function both in the viral replication process and in innate immunity. The NS3 protease has a catalytic triad composed by His51/Asp75/Ser135 residues, located in a cleft between the *β*-barrels [2]. The NS2B protein acts as a cofactor of the NS3 protease, undergoing a conformational change during the binding to NS3, necessary to activate the protease [7]. NS2B is a large membrane protein of 130 amino acids and consists of three hydrophobic domains and a central hydrophilic domain, where its *C*-terminal portion is responsible for the recognition site [8]. NS2B/NS3 protease is also implicated in immune invasion through cleavage of the human mediator of activation of interferon regulatory factor-3 activator (IRF-3) [9].

**Figure 1:**
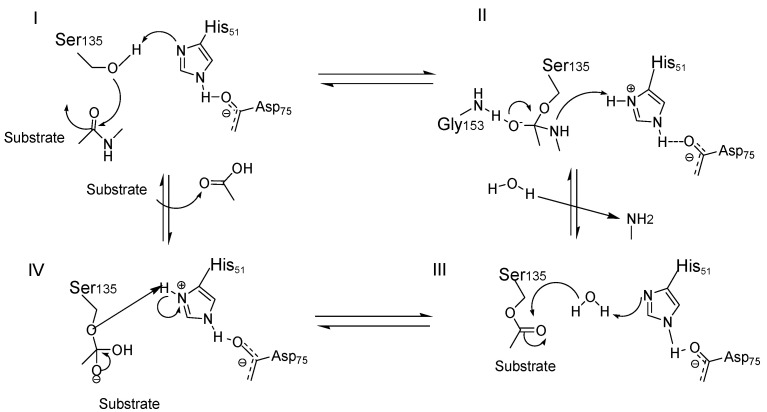
Catalytic cycle of the serine protease NS3pro has a functional catalytic triad comprising the His51, Asp 75, and Ser135 amino acid residues. Then, the cleavage of peptidic substrates begins by a Ser135 -nucleophilic attack to the carbonyl group at the P1 position. Due to the inherent poor nucleophilicity of the hydroxyl group from the Ser135, it should be previously activated by the action of an adjacent His51 residue, generating the Ser135–O− nucleophile (I). Subsequently, the stabilization of this complex into the oxyanion hole via H-bond interactions with Gly 153 residue favors the formation of a tetrahedral intermediary (II). This tetrahedral state is decomposed and results in the C-terminal cleavage, releasing an amine fragment. The N-terminal fragment remains covalently connected to the protease via an ester bond, which is posteriorly hydrolyzed by the action of a water molecule. In this step, His 51 acts as a base in order to increase the nucleophilic character of this water molecule (III). Finally, the N-terminal fragment is released by reprotonation of the carboxylic acid, beginning a new catalytic cycle (IV).

The cleavage of the protease involves the nucleophilic attack of the Ser135–O-nucleophile, generated by His51 basic catalysis, on the carbonyl group at the P1 position, generating the tetrahedral intermediate stabilized in the oxyanion hole via H-bond interactions with Gly153 residue (Figure 1-I). This tetrahedral intermediate decomposes and results in *C*-terminal cleavage, releasing an amine fragment (Figure 1-II). Differently, the *N*-terminal fragment remains covalently connected to the protease via an ester bond, which is hydrolyzed by a water molecule. At this point, His51 acts as a base in order to increase the nucleophilic character of the water molecule (Figure 1-III), and therefore, the *N*-terminal fragment is released by deprotonation of the carboxylic acid, leading to the beginning of a new catalytic cycle (Figure 1-IV) [2].

NS3 is also one of the major viral proteins showing an enzymatic function; it is a 69 kDa protein, and it possesses two main domains with different enzymatic functions: a trypsin-like serine protease domain situated within the *N*-terminal with 180 amino acid residues, while the *C*-terminal domain has the activities of an RNA-helicase [2]. Due to these functions, the NS2B/NS3 serine protease represents a promising target for the development of new agents for the treatment of DENV infections.

ZIKV is a mosquito-borne infection, and the principal way of transmission is through mosquito bites by *Aedes aegypti* and *Aedes albopictus*. The ZIKV contains a +ssRNA genome, with about 10800 nucleotides, that encodes for a precursor polyprotein that is processed by proteases into the three structural proteins, i.e., the capsid (C), the premembrane/membrane (prM), and the envelope protein (E), and seven non-structural proteins (NS1, NS2A, NS2B, NS3, NS4A, NS4B, and NS5) [5].

The ZIKV NS3 is a multifunctional protein with two functionally distinct domains: a 176-residue *N*-terminal domain with protease activity and a 444-residue *C*-terminal domain with helicase, nucleoside 5′-transferase (NTPase), and 5′-terminal RNA triphosphatase (RTPase) activities. The NS2B polypeptide cofactor plays a key role, like for DENV, for NS3 catalysis. 

The ZIKV NS2B/NS3 sequence shows high homology with other flavivirus proteases including DENV NS2B/NS3; the main difference consists in two residues: Glu/Ala 153 and Lys/Asp 139 in DENV and ZIKV proteases, respectively [10].

ZIKV NS3 is a serine protease containing a catalytic triad of serine, histidine, and aspartate (His51/Asp75/Ser135) in its binding site, and it requires NS2B as cofactor domain [11]. In the active form, the *C*-terminal part of NS2B wraps around the active site of NS3, so that it could form a *β*-hairpin to create the S2 and S3 pockets of NS3 protease [11]. Many studies from the literature showed that the protease can adopt two conformations: “open” and “closed”; in the closed state that is catalytically active, NS2B is fully bound around NS3 in the active site, while in the “open” state, that is the inactive conformation, NS2B is partially bound to NS3 [12]. In the closed conformation, the NS2B wraps around NS3 as the active form, while in the “open conformation”, the NS2B chain turns and binds the portion behind the active site, thus inducing its inactivation [13].

## 3. Interaction of Flavivirus NS2B/NS3 with Cellular Proteins

The flaviviral RNA tends to replicate on the membrane of the replication site for DENV, leading to the constitution of a replication complex, where NS2B/NS3 takes part in the maintenance of the same complex. The DENV NS3 protein redirects the fatty acid synthase; in fact, it has been demonstrated that DENV-infected cells showed an increased synthesis of fatty acids during infection [14]. 

Furthermore, it was reported that the DENV NS3 interacts with glyceraldehyde-3-phosphate dehydrogenase (GADPH), thus leading to an increased ATPase activity and to a reduced glycolytic activity. Also, the interaction between NS3 and GADPH may result in the unwinding of double-stranded RNA and in the vesicle formation needed for virion assembly [15,16,17]. 

Currently, it seems that the NS3 proteases of DENV and ZIKV caused the cleavage of FAM134B (a host cell restriction factor involved in cells in the process of reticulophagy), thus leading to an enhanced viral replication [18].

The ZIKV NS2B/NS3 protease is also implicated in interactions with many other cellular proteins, which include the cleavage of the cytoskeletal factor, septin-2, resulting in slow cell division, enhanced apoptosis, and delayed cytokinesis in the neural progenitor cells (NCPs). These modifications produced microencephalopathy [19]. Mitochondrial-associated membranes (MAMs) are also known to play an important role in several processes that are crucial for viral replication; so, DENV NS2B/NS3 protease interacts with mitochondria and results in the cleavage of MAMs, leading to the fragmentation of the mitochondria, which contributes to disease pathogenesis [20].

## 4. Crystal Structures of DENV and ZIKV NS2B/NS3 Protease

Several crystal structures of flavivirus proteases in the presence or in the absence of inhibitors were determined [21,22,23,24,25,26,27,28,29]. All these structures are based on proteins obtained by recombinant DNA missing from the NS2B transmembrane domains, and the folding of full-length NS2B requires the presence of detergent micelles as membrane systems [30,31,32,33]. For DENV protease, a cofactor region of about 40 residues taken from NS2B and NS3 protease domain (NS3pro) connected via a glycine-rich linker was taken into consideration in the structural studies [34].

In DENV and ZIKV NS2B/NS3 protease structures, the folds of NS3 in several X-ray structures are almost identical. The *N*-terminal domain of NS3 is a serine protease containing two *β*-barrels, and each barrel consists of six *β*-strands. The catalytic triad is composed by His, Asp, and Ser residues and is totally conserved among these proteases [21]. Considering that the active site is negatively charged, this favors molecular interactions with positively charged residues, for example, Lys and Arg (Figure 1) [35].

The structures of free DENV NS2B/NS3 exist in an open inactive conformation [28,29,36], in which the *C*-terminal region of the NS2B cofactor is positioned away from the active site. The amino acids of the *C*-terminal portion of NS2B form a *β*-hairpin structure through molecular interactions with the substrate [37,38].

On the contrary, the crystal structure of free ZIKV NS2B–NS3 protease reveals that the protease adopts the closed active conformation [24], even though this conformation might be observed in the X-ray studies due to crystal packing. Several NMR studies demonstrated that the closed conformation of the protease is predominant in the solution and should be used in structure-based drug design [39].

## 5. NS2B/NS3 Protease Inhibitors

### Allosteric Inhibitors 

**Figure 2:**
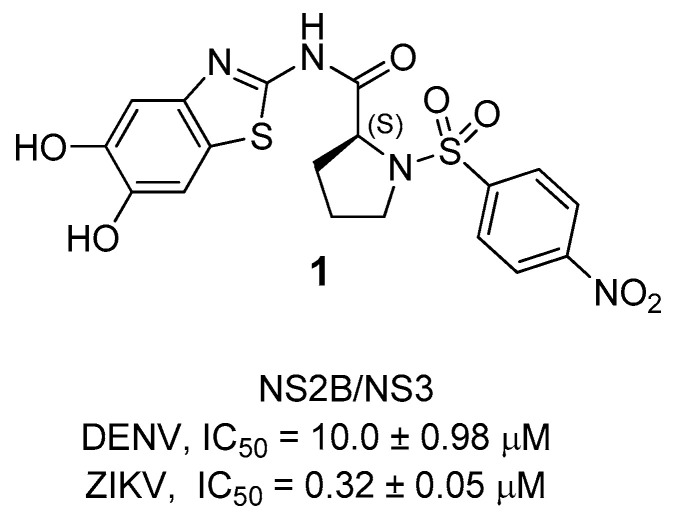
Structure of the lead compound **1**.

Starting from the lead compound **1**, a library of new proline-based inhibitors was tested on ZIKV and DENV NS2B/NS3 protease [40]. The lead compound **1** was the first inhibitor reported; it shows a 2-aminobenzothiazole ring linked to a proline residue, functionalized with a 4-nitrophenylsulsulfonyl moiety. Starting from its structure, several structural changes were carried out with the aim to improve the antiviral activity (Figure 2) [40].

**Figure 3:**
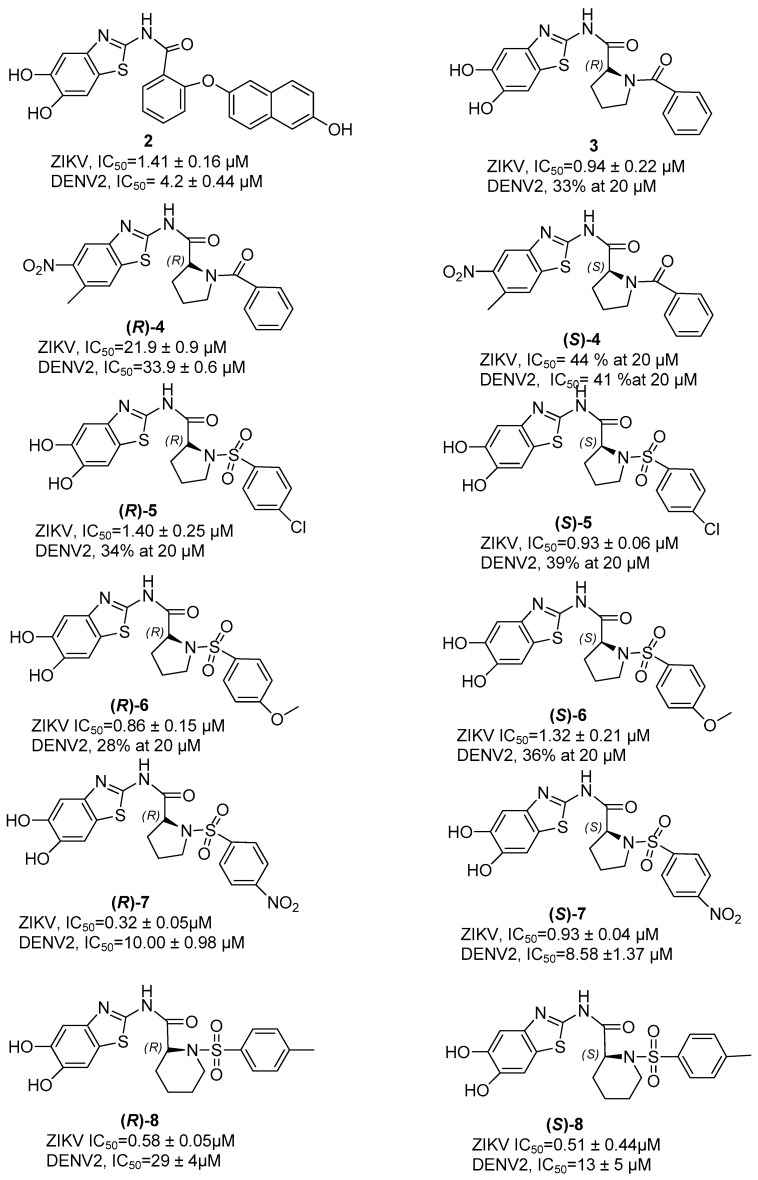
Structure and activity against ZIKV and DENV2 proteases of proline-based allosteric inhibitors **2**–**8**.

In compound **2** (Figure 3), the proline has been replaced by a 2-hydroxybenzoic acid that establishes an ether bridge with a hydroxyl-substituted naphthalene ring. This compound shows activity against DENV and ZIKV proteases expressed by IC_50_ values of 4.2 ± 0.44 µM and 1.41 ± 0.16 µM, respectively. 

Thus, maintaining the unchanged benzothiazole moiety, the benzamide portion was replaced with a *R* configured proline residue, where the amino acid NH was benzoyl-substituted, thus obtaining compound **3** endowed of a slight increase in activity against ZIKV (IC_50_ = 0.94 ± 0.22 µM), while it is inactive against DENV [40]. On the contrary, as shown in compound **4**, the replacement of the hydroxyl group with the nitro group leads to a general decrease in activity. In this molecule, we can observe a decrease in the activity due to the substitution of the OH groups both in the case of the *R*-enantiomer of proline (ZIKV IC_50_ = 21.9 ± 0.9 µM and DENV IC_50_ = 33.9 ± 0.6 µM) and for the *S*-enantiomer of proline of the compound **4** (ZIKV IC_50_ = 44% and DENV IC_50_ = 41%). 

Other active proline-based inhibitors are compounds **5** and **6** in which the *N*-benzoyl substituent has been replaced with a chloro- or methoxy-substituted phenyl sulfonyl group, respectively, thus obtaining the best results with (*S*)-**5** endowed with an IC_50_ of 0.93 ± 0.06 µM and (*R*)-**6** with an IC_50_ of 0.86 ± 0.15 µM against ZIKV protease, respectively. 

Then, in compounds (*R*)-**7** and (*S*)-**7**, a nitro group was introduced on the phenyl ring linked to the proline residue via a sulfonyl bridge, investigating at the same time the role of the configuration on the proline residue [40].

The results of this structure–activity relationship (SAR) investigation clearly show that the introduction of the nitro group is fruitful, since a slight increase in the antiviral activity has been observed (ZIKV, IC_50_ = 0.86 ± 0.15 µM for (*R*)-**6** versus 0.32 ± 0.05 µM for (*R*)-**7**). 

Concerning the role of the stereochemistry at the proline residue, an improved activity of the *R*-enantiomer with respect to the *S*-counterpart in ZIKV was observed, while in the case of DENV, the *S*-enantiomer resulted to be most active. 

Inhibitors based on the replacement of proline with a piperidine moiety in both *R* and *S* configurations were also developed (e.g., compounds (*R*)-**8** and (*S*)-**8**, Figure 3). The results of this investigation clearly highlight that in the case of ZIKV protease, piperidine is preferred to proline, with an improved activity for the *S*-enantiomer. Differently, in the case of DENV protease, the *S*-configured piperidine gave better results. 

**Figure 4:**
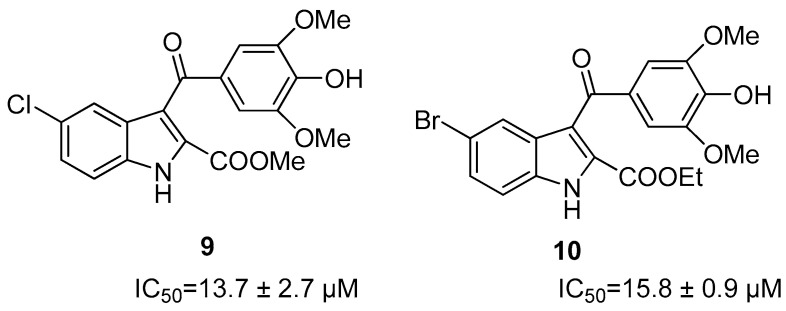
Structure of allosteric inhibitors without proline **9**–**10**.

Subsequently, allosteric inhibitors without a proline residue were developed, like compounds **9** and **10** (Figure 4). Both share the presence of an indole ring linked via a carbonyl group to an aromatic ring bearing two methoxy and a hydroxy group. Compound **9** presents an indole ring, where there is a COOMe group at position 2, while at position 5 a chlorine atom, and the heterocycle is connected via a carbonyl group to an aromatic ring that bears two methoxy and a hydroxyl group. On the contrary, compound **10** differs from compound **9** due to the presence of a bromine instead of a chlorine [40]. When tested against NS2B/NS3 protease, the most active was compound **10**, with an IC_50_ value against ZIKV protease of 33 µM compared to compound **9**, whose IC_50_ value is 15.8 ± 0.9 µM. These inhibitors were, in addition, tested on ZIKV-infected Huh-7 cells, where it was observed that the most active compound was **9**, with an EC_50_ value of 13.9 ± 0.4 µM compared to that of compound **10**, whose EC_50_ value was 16.2 ± 0.6 µM.

According to the Lipinski’s rule of five, these two inhibitors were predicted to have a good oral absorption. In a mouse model of ZIKV infection, it has been demonstrated that compound **9** prevents brain damage caused by the viral infection [40]. 

**Figure 5:**
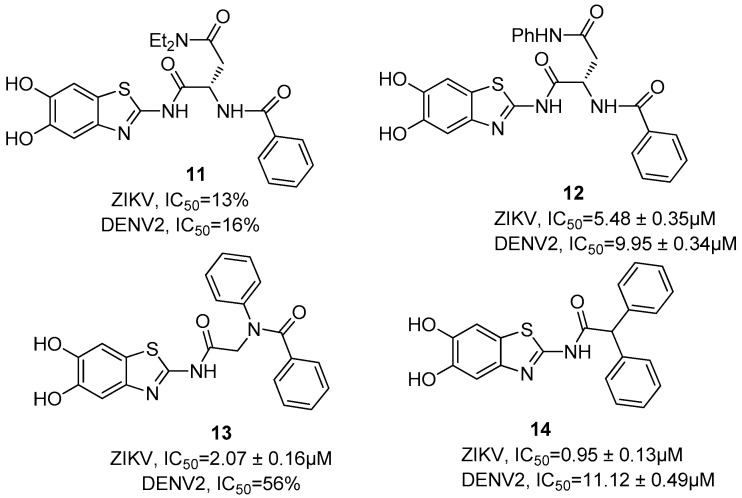
Structure and activity against ZIKV and DENV2 proteases of allosteric inhibitors **11**–**14**.

Further allosteric inhibitors, without a proline residue, were designed and synthesized; some of these are asparagine-based inhibitors. These structures show the common presence of the 2-amino-5,6-dihydroxybenzothiazole moiety, functionalized at the 2-NH_2_ group with an *N*,*N*-diethyl-substituted (i.e., **11**, Figure 5) or *N*-phenyl-substituted (i.e., **12**, Figure 5) asparagine [41].

Compound **11** showed a very low inhibition at 20 µM (13% for ZIKV and 16% for DENV2) of NS3/NS2B, whereas the corresponding aniline derivate **12** led to an improvement in the antiviral activity, with IC_50_ values of 5.48 ± 0.35 µM and 9.95 ± 0.34 µM for ZIKV and DENV, respectively. 

**Figure 6:**
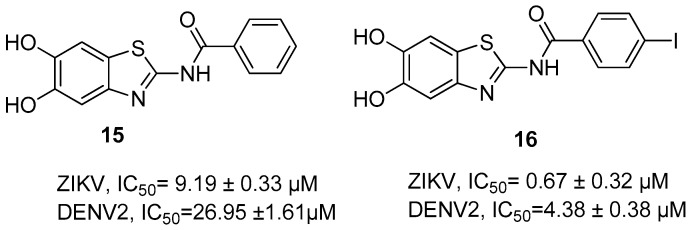
Allosteric inhibitors of the DENV and ZIKV NS2B/NS3 proteases.

In this situation, aromatic substituents seem to be preferred; thus, inhibitor **13** (Figure 5) incorporating a rigid *N*-phenyl peptoid structure showed a slight decrease in activity against DENV NS3/NS2B (56% of inhibition at 20 µM) and an improved antiviral activity towards ZIKV (IC_50_ = 2.07 µM), whereas compound **14** (Figure 6) that contains a 2,2-diphenylacetic acid showed a submicromolar IC_50_ value against ZIKV (IC_50_ = 0.95 ± 0.13 µM) and an IC_50_ value in the micromolar range against DENV protease (IC_50_ = 11.12 ± 0.49 µM).

Further allosteric inhibitors, without a proline residue, were developed with the aim to improve the antiviral activity, and the selectivity towards DENV and ZIKV proteases was consistent for all tested inhibitors, based on the nature of the various substituents [41].

All these compounds are characterized by the presence of a benzothiazole ring variously decorated with hydroxyl groups; this nucleus, by means of an amide or a thiourea bond, binds to an aromatic ring with various substituents such as iodine, chlorine, methyl atoms, etc.

**Figure 7:**
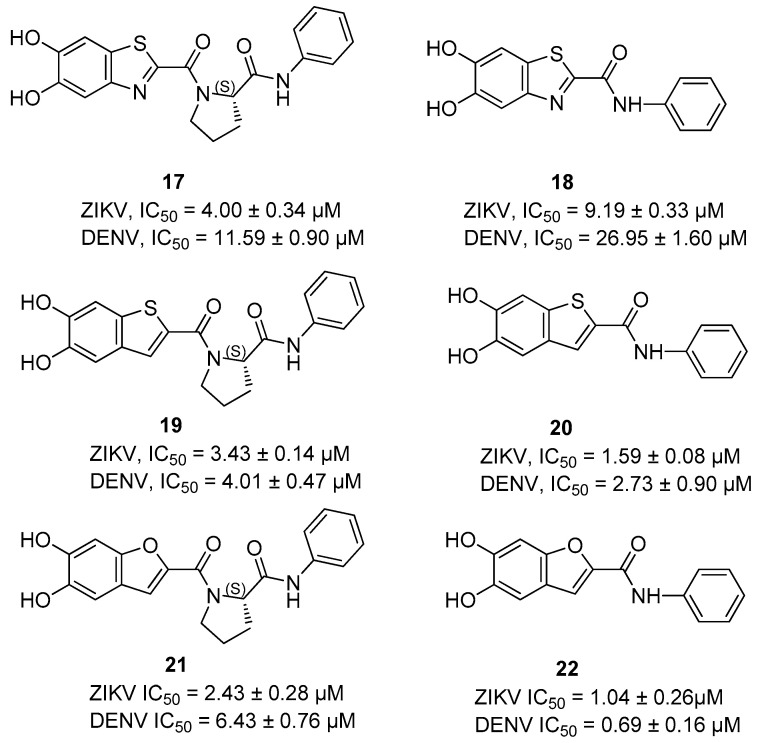
Allosteric inhibitors of the DENV and ZIKV NS2B/NS3 proteases **17**–**22**.

Compound **15** (Figure 6) that has no substituents on the aromatic ring showed an activity in the micromolar range for ZIKV and DENV proteases (IC_50_ = 9.19 ± 0.33 µM and IC_50_ = 26.95 ± 1.61 µM, respectively). Among all the substitutions on the phenyl ring, the best one is the insertion of an iodine atom, i.e., **16** (Figure 7), which led to a submicromolar activity against ZIKV (IC_50_ = 0.67 ± 0.32 µM) and to a micromolar activity against DENV2 (IC_50_ = 4.38 ± 0.38 µM, respectively). 

As the SAR optimization strategy, further replacements were made, leading to a variety of inhibitors characterized by a heterocyclic structure, which were evaluated for their inhibitory properties towards the NS2B/NS3 proteases [41].

These compounds share the presence of a heterocycle connected via a carbonyl group to a linker that binds an aniline, where the linker of these structures could be a proline residue or a pipecolic acid (Figure 7). Compound **17** bears two hydroxyl groups on the benzothiazole ring linked via a carbonyl group to a (*S*)-proline residue that binds a benzamide substituent. The compound **18** differs from compound **17** due to the lack of a proline residue, and the results of this investigation clearly highlight that this linker is crucial for the inhibitory properties. 

The replacement of the benzothiazole ring with a benzothiophene nucleus, i.e., **19**–**20**, led to an increase in the antiviral activity. However, the best replacement of the benzothiazole nucleus was proven to be the benzofuran ring, which led to the most active inhibitors of the NS2B/NS3 protease. 

**Figure 8:**
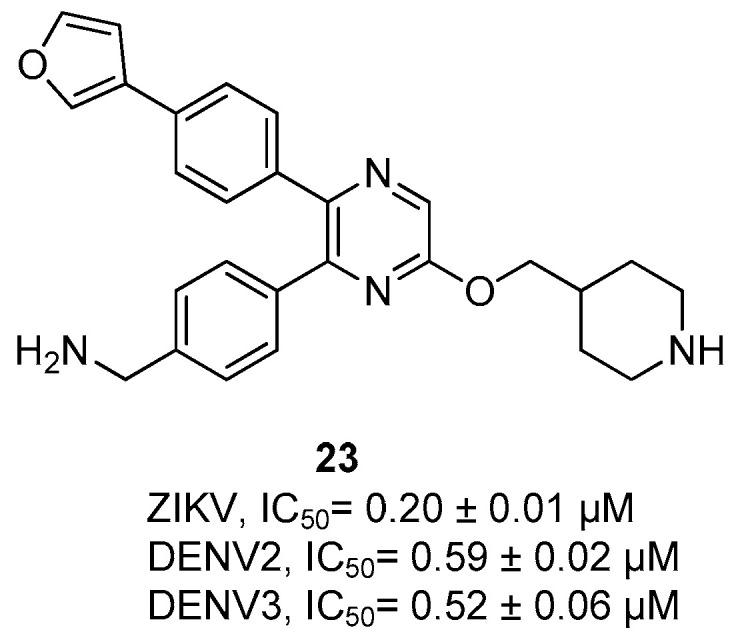
Structure and activity against ZIKV and DENV2 proteases of the pyrazine-based allosteric inhibitor **23**.

Compound **23** (Figure 8) is an allosteric inhibitor without a proline residue, and it was shown to be a broadly active inhibitor of flavivirus proteases endowed with a high selectivity [12]. Its structure is completely different with respect to the previously described compounds, and in fact, it presents a pyrazine connected via an ether bridge to a piperidine nucleus, and furthermore, the pyrazine nucleus is characterized by the presence of two substituents, i.e., at the position 5 a 4-phenyl methyl amino substituent and at the position 6 a 4-(furan-3-yl) phenyl group. Compound **23** resulted to be a broad spectrum flavivirus NS2B/NS3 protease inhibitor since it inhibited the serine protease of ZIKV and DENV serotype-2 and 3 (ZIKV IC_50_ = 0.20 ± 0.01 µM, DENV2 IC_50_ = 0.59 ± 0.02 µM, DENV3 IC_50_ = 0.52 ± 0.06 µM) [12]. 

Inhibitor **23** exhibited a significant in vivo activity, since when administered in ZIKV-infected C57BI/6 mice, it was able to reduce 98% of ZIKV RNA copies in both plasma and brains, thus inhibiting its replication in vivo. 

## 6. Orthosteric Inhibitors

### Dengue NS2B/NS3 Protease Inhibitors

Orthosteric inhibitors are a class of active compounds against DENV and ZIKV NS2B/NS3 proteases that bind to the active site of the enzyme, differently from the allosteric inhibitors [27,36,42,43,44]. All inhibitors bear two basic residues (arginine, lysine, or a mimetic) to address the dibasic substrate recognition motif [2,45]. 

The first inhibitors reported in the literature were developed as covalent ligands and are characterized by the presence of two basic aminoacidic residues linked to an electrophilic moiety, such as trifluoromethyl ketone, [46] aldehyde, [47], and boronic acid [48], able to covalently trap the catalytic serine. However, if ligands do not show a reactive warhead, they can non-covalently bind to the active site. 

**Figure 9:**
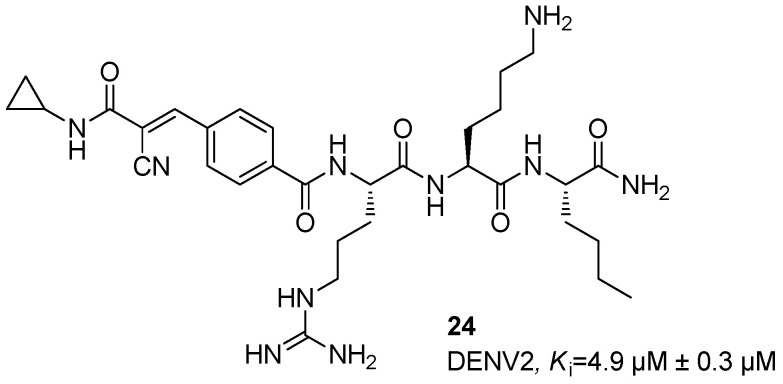
Retro-tripeptide inhibitor **24**.

Initially, some inhibitors based on α-ketoamide and arylcyanoacrylamide warheads were synthesized [49,50,51]. Starting from *α*-ketoamides and arylcyanoacrylamides, several studies were carried out [52,53], by investigating retro, retro-inverse, semi retro-inverse, and non-retro inverse peptides, thus identifying as the most promising peptide the retro-tripeptide **24**, characterized by the presence of a cyanoacrylamide group at the aromatic ring of the *N*-benzoyl capped Arg-Lys-Nle-NH_2_ and endowed with a *K*_i_ value of 4.9 ± 0.3 µM against DENV2 NS2B/NS3 protease (Figure 9). Furthermore, the Nle amino acid residue was verified to be crucial for the selectivity of this compound.

**Figure 10:**
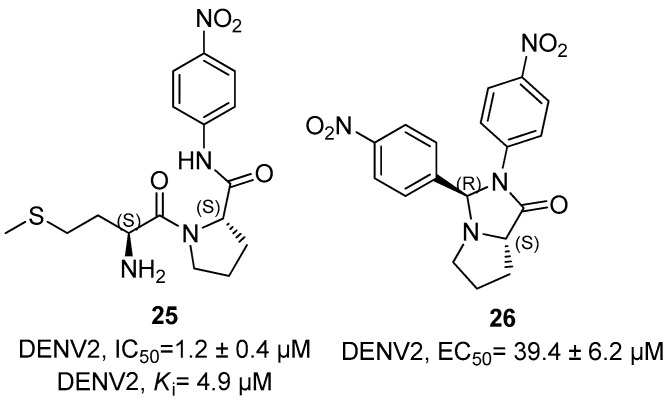
Met-Pro dipeptide inhibitor **25** and its fused-bicyclic derivate **26**.

Dipeptides **25** and **26** were further developed against NS2B/NS3 protease of DENV2; among these, compound **25** characterized by the Met-Pro sequence showed an excellent activity of anti-DENV2 with IC_50_ and *K*_i_ values of 1.2 ± 0.4 and 4.9 µM (Figure 10), while compound **26** showing a fused-bicyclic pyrrolidine moiety showed an EC_50_ value against DENV2 NS2B/NS3 protease in the middle micromolar range (i.e., EC_50_ = 39.4 ± 6.2 µM, Figure 10) [54,55]. 

**Figure 11:**
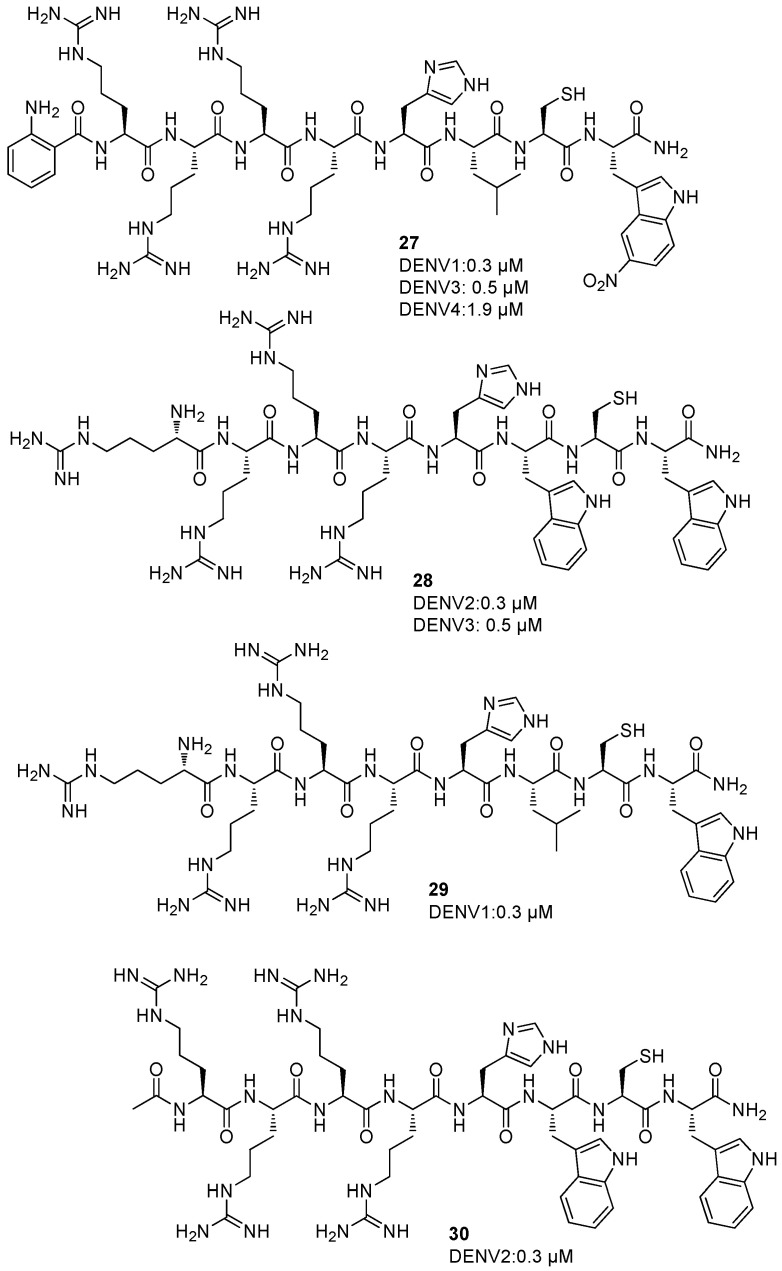
Peptidomimetics against DENV 1-4 NS2B/NS3 protease **27**–**30**.

Several peptidomimetics characterized by a characteristic peptide sequence were screened against DENV 1-4 NS2B/NS3 protease to evaluate the binding affinities (*K*_i_) [56,57]. These inhibitors showed good activities against DENV; compound Abz-Arg-Arg-Arg-Arg-His-Leu-Cys-Trp-Tyr(NO_2_)-NH_2_ (**27**) revealed a good activity towards DENV1 (IC_50_ = 0.3 µM), DENV3 (IC_50_ = 0.5 µM), and DENV4 (IC_50_ = 1.9 µM) NS2B/NS3 protease. The peptidomimetic H-Arg-Arg-Arg-Arg-His-Trp-Cys-Trp-NH_2_ (**28**) showed an excellent activity against DENV2 and DENV3 NS2B/NS3 protease, with *K*_i_ values of 0.3 and 0.5 µM, respectively (Figure 11). Moreover, compound H-Arg-Arg-Arg-Arg-His-Leu-Cys-Trp-NH_2_ (**29**) revealed to possess a good activity against DENV1 NS2B/NS3 protease, with a *K*_i_ value of 0.3 µM (Figure 11). Finally, compound Ac-Arg-Arg-Arg-Arg-His-Trp-Cys-Trp-NH_2_ (**30**, Figure 11) also presented a good activity against DENV2 NS2B/NS3 protease, with a *K*_i_ value of 0.3 µM. 

Docking studies put in evidence that the *N*-terminal region of all these compounds interacts with Asp75 in the catalytic site, the cysteine residue is turned towards His51 and Ser135, and the two P1 and P2 residues occupy the S3 and S4 pockets. 

**Figure 12:**
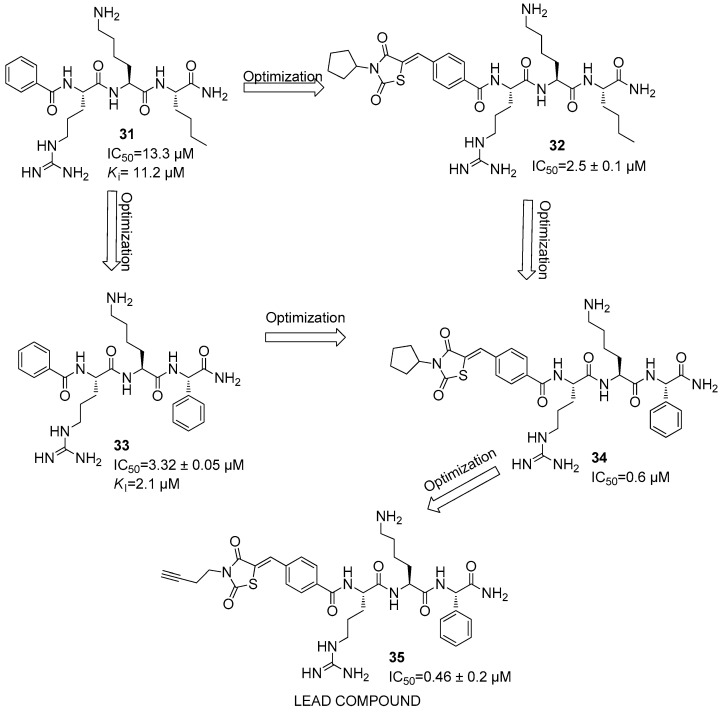
Structure of inhibitors **31**–**35**.

After several studies, further promising inhibitors were developed against the DENV2 NS2B/NS3 protease [58]. These compounds are characterized by a backbone of three amino acids coupled with a non-peptidic *N*-terminal group, i.e., a benzoyl group, thus leading to compound **31**, which showed IC_50_ and *K*_i_ values of 13.3 µM and 11.2 µM (**31**) (Figure 12). 

However, the most active peptide-hybrid was constituted by an Arg-Lys-Nle-NH_2_ sequence capped with a 2,4-thiazolidinedione moiety, thus showing an IC_50_ value of 2.5 ± 0.1 µM (i.e., **32**) (Figure 12). 

Furthermore, Ref. [59] it was observed that the replacement of *C*-terminal Nle residue in compound **31** with other residues like phenylglycine (Phg) led to an increase in the activity of the parent compound **31**. Inhibitor **33** showed IC_50_ and *K*_i_ values of 3.32 ± 0.05 and 2.1 µM, respectively, against DENV2 NS2B/NS3 protease (Figure 12). Additionally, the Phg analogue of compound **32** showed an IC_50_ value of 0.6 µM against DENV2 (**34**) (Figure 12). Docking studies put in evidence that the Phg residue interacts with residues in the S1 pocket, while Arg and Lys interact with the S2 and S4 pockets, respectively [60].

**Figure 13:**
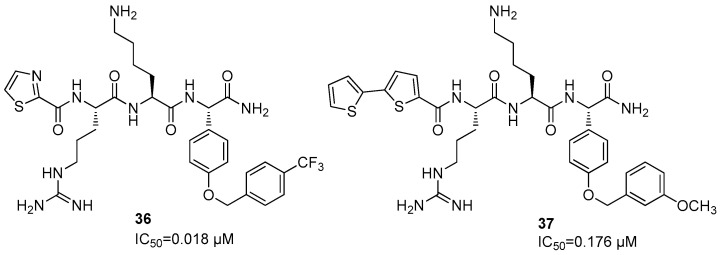
Structure of inhibitors **36** and **37**.

A further SAR investigation led to the synthesis of compound **35**, which bears a butynyl group linked to the 2,4-thiazolidinedione nucleus and showed the best IC_50_ value for this series of compound (IC_50_ = 0.46 ± 0.2 µM versus 0.6 µM for **35** and **34**, respectively) (Figure 12). Docking studies revealed that Phg is located in the S1 pocket, and Arg residue in the S2. A further SAR investigation was carried out by synthesizing thiazolidinylcarbonyl-Arg-Lys-(OCH_2_C_6_H_6_(4-CF_3_)-Phg-NH_2_ (**36**) and bis-thiophenylcarbonyl-Arg-Lys-(OCH_2_C_6_H6(_3_-OCH_3_)-Phg-NH_2_ (**37**), which show IC_50_ values of 0.018 and 0.176 µM, respectively (Figure 13).

Both compounds were able to interact with His51, Asp75, and Ser135 amino acid residues of the catalytic triad in the binding site [61,62]. 

**Figure 14:**
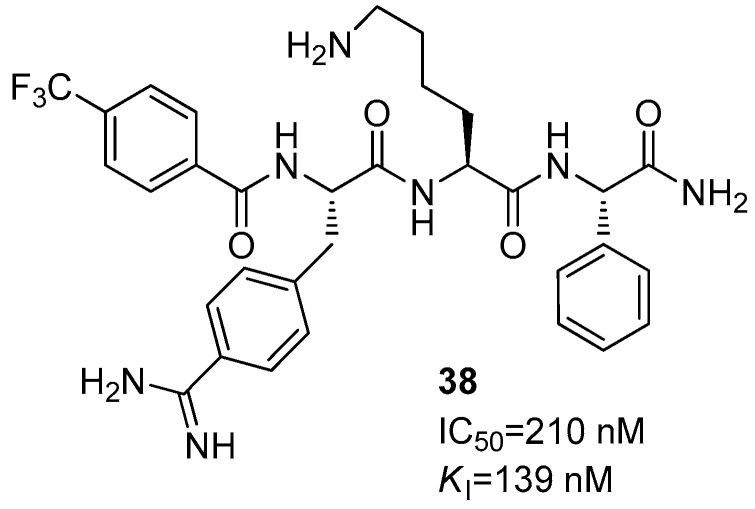
Peptide-hybrid inhibitor **38** of DENV2.

Another structural variation has been carried out by introducing in compound **33** a 3-trifluoromethyl benzoyl cap to the *N*-terminal amino acid and by switching the P3 Arg residue with a 4-amidino Phe, leading to compound **38** with IC_50_ and *K*_i_ values of 210 and 139 nM, respectively (Figure 14) [62] The new basic residue accommodates into the S2 pocket and interacts with Asp75 through electrostatic interactions [63].

**Figure 15:**
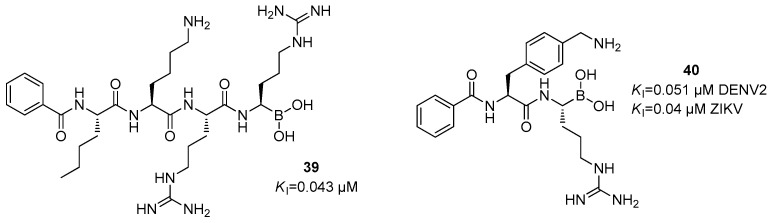
Peptidomimetics **39** and **40** containing a boronic acid as a warhead.

Another class of orthosteric inhibitors is represented by peptide boronates, among which the most active inhibitor is Bz-Nle-Lys-Arg-Arg-B(OH)_2_ (**39**) that shows a *K*_i_ value of 0.043 µM against the viral protease (Figure 15). 

The introduction of this new electrophilic warhead led to an increase in inhibitory potency, being responsible of a 1000-fold enhancement in affinity. Another dipeptide endowed with the boronic acid moiety is Bz-(4-CH_2_NH_2_)Phe-Arg-B(OH)_2_ (CN-716, **40**), which showed *K*_i_ values of 0.051 and 0.04 ± 0.06 µM against DENV2 and ZIKV proteases, respectively (Figure 15) [27].

Successively, it was observed that aldehydes also can interact with the nucleophile serine residue of the NS2B/NS3 protease in a covalent-reversible mode [47,49].

**Figure 16:**
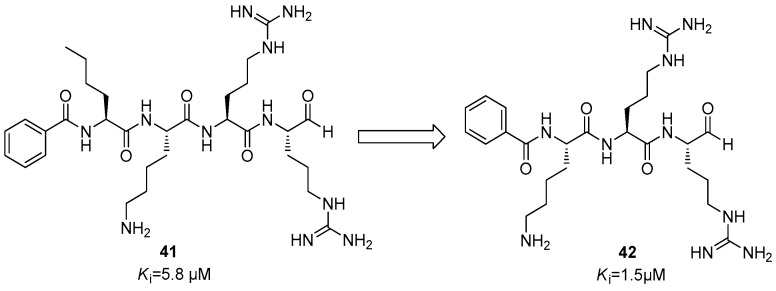
Inhibitors endowed with an aldehyde warhead **41** and **42**.

The first synthesized compound was Bz-Nle-Lys-Arg-Arg-H (**41**) (Figure 16), which showed a *K*_i_ value of 5.8 µM. Starting from this compound, through an optimization process by molecular simplification, Bz-Lys-Arg-Arg-H (**42**) was obtained that showed an improved inhibitory activity with a *K*_i_ value of 1.5 µM (Figure 16) [64,65].

**Figure 17:**
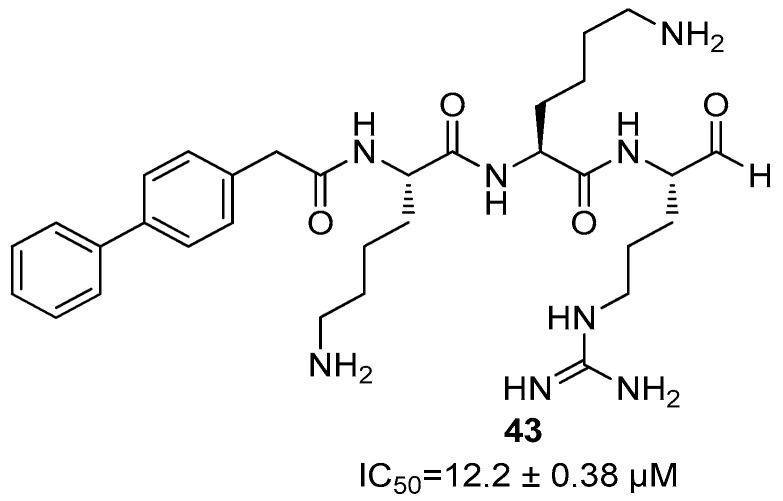
Inhibitor **43** endowed with an aldehyde warhead.

Lastly, the introduction of a 4-biphenylacetyl group linked to the *N*-terminal of a lysine residue led to compound **43**, with the sequence 4-biphenylacetyl-Lys-Lys-Arg-H, which showed an IC_50_ value in the mid-micromolar range (IC_50_ = 12.2 ± 0.38 µM, Figure 17). 

**Figure 18:**
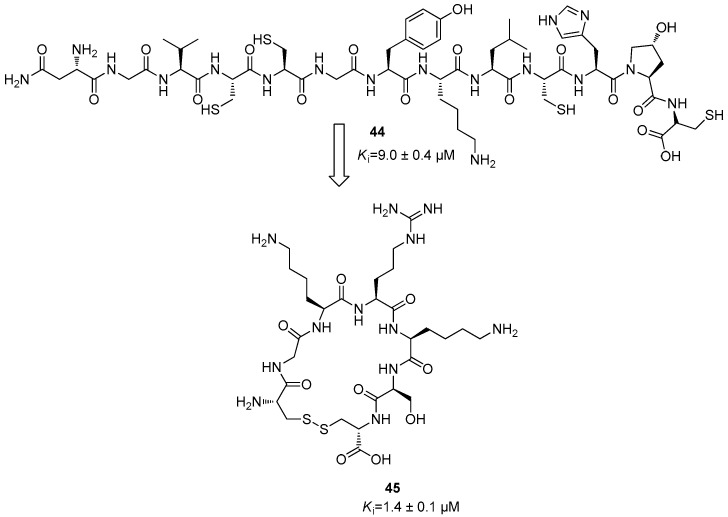
Structure of polypeptide **44** and of the cyclic peptide **45**.

Furthermore, additional libraries of cyclic peptides were designed to efficiently interact with the orthosteric site of the NS2B/NS3 protease. Some of these compounds were derived from the conotoxin class produced by *Conus* species. These conotoxins are composed by a mixture of neurotoxins produced by a snail; so, it seemed that MrIA conotoxin presents an interesting inhibitory activity with a *K*_i_ value of 9.0 ± 0.4 µM against DENV2 NS2B/NS3 [66,67]. This toxin is constituted by a 13-residue sequence H-Asn-Gly-Val-Cys-Cys-Gly-Tyr-Lys-Leu-Cys-His-Pro(OH)-Cys-OH (**44**) (Figure 18); after several structural modifications, it was observed that the seven-residue sequence of the cyclic peptide c(Cys-Gly-Lys-Arg-Lys-Ser-Cys) (**45**) represented the most active compound of this series, with a *K*_i_ value of 1.4 ± 0.1 µM. (Figure 18) [68].

**Figure 19:**
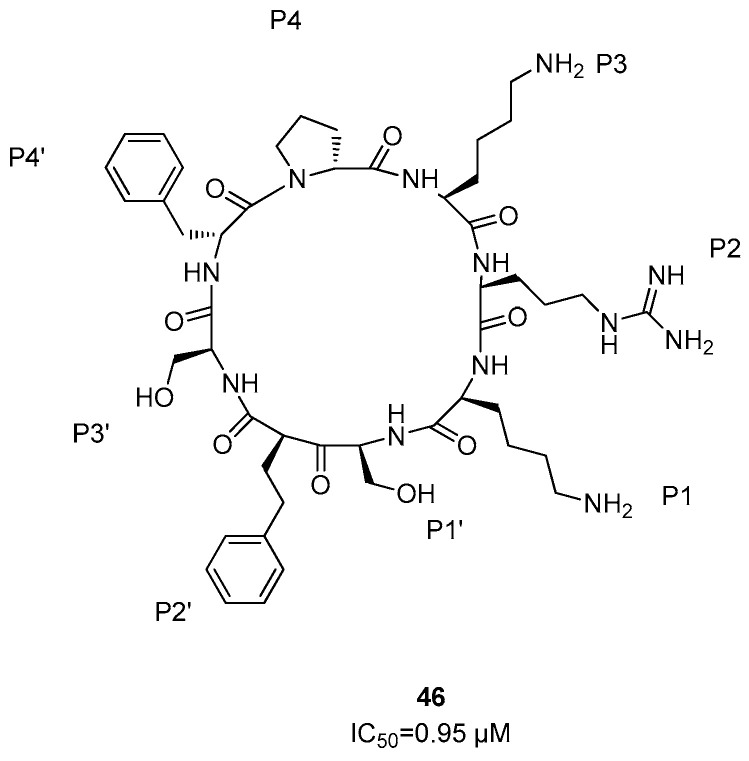
Cyclic peptide **46**.

Cyclic peptides represent a chemical class of molecules able to interact with biomacromolecules by protein–protein interactions [69,70]; among these, the macrocyclic peptidomimetic compound (**46**) constituted by the amino acid sequence D-Pro-L-Lys-L-Arg-L-Lys-L-Ser-L-Phe-L-Ser-D-Phe (i.e., **46**) was demonstrated to be the most active derivative, with an IC_50_ value of 0.95 µM (Figure 19) [71]. It was observed that the side chains of this compound interact with hydrogen bonds with Asp129 of NS3 protease and with Asp81 and Met84 of NS2B, respectively.

**Figure 20:**
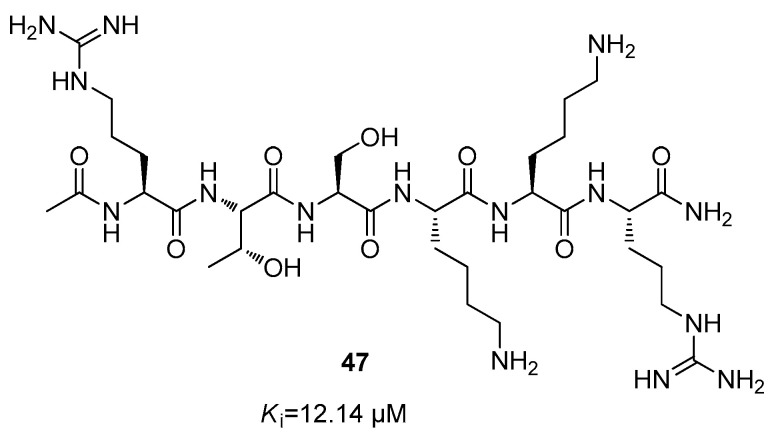
Structure of inhibitor **47**.

Further, it was reported in the literature that NS3 protease of Hepatitis C virus (HCV) was moderately inhibited by *N*-terminal peptides derived from polyprotein cleavage sites [72,73]. Therefore, a potent inhibitor towards DENV NS3 protease was developed with an IC_50_ value in the micromolar range, showing the peptide sequence Ac-Arg-Thr-Ser-Lys-Lys-Arg-NH_2_ (**47**) (Figure 20) [74]. 

Aprotinin (AP) is a small protein developed as an inhibitor of bovine pancreatic trypsin (BPTI); it was a potent inhibitor of NS2B/NS3 protease from DENV and ZIKV at the nanomolar level. Furthermore, starting from the complex reported in the PDB Aprotinin-NS2B/NS3 protease (PDB code 3U1J), a new series of cyclic peptides has been developed [75]; however, the binding loop (BL) of the aprotinin structure was constituted by seven residues Pro13-Cys14-Lys15-Ala16-Arg17-Ile18-Ile19. 

## 7. Zika NS2B/NS3 Protease Inhibitors

**Figure 21:**
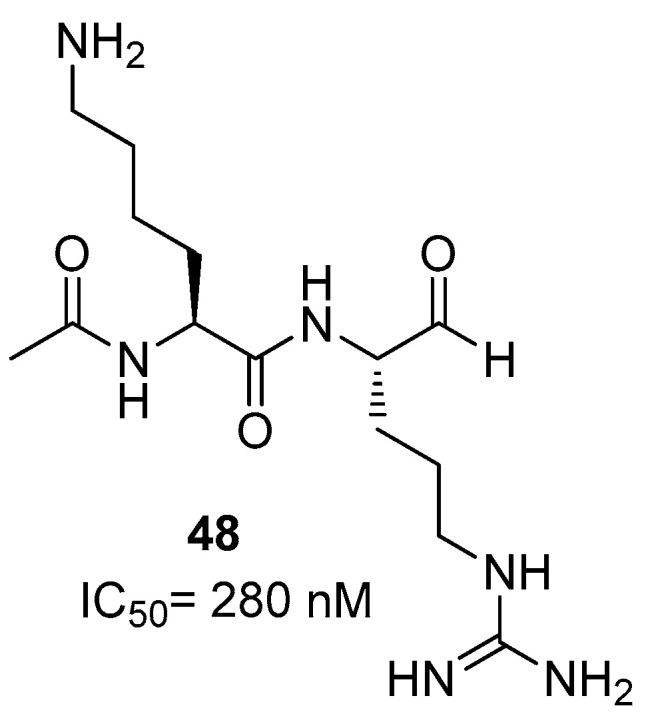
Peptide aldehyde inhibitor **48** of NS2B/NS3 protease from ZIKV.

Concerning the development of ZIKA NS2B/NS3 protease inhibitors, a potent class is represented by peptides bearing an aldehyde warhead. Among all the synthesized compounds, inhibitor **48** was demonstrated to interact using a covalent mode with Ser135 residue of NS3 protease, with an IC_50_ value of 280 nM (Figure 21) [76]. Docking studies showed that P2 Lys and P1 Arg residues are located in the S2 and S1 sites of the protease, while Ser135 is covalently bonded to the carbonyl group of the aldehyde and His51 binds to the molecular complex via H-bond. 

**Figure 22:**
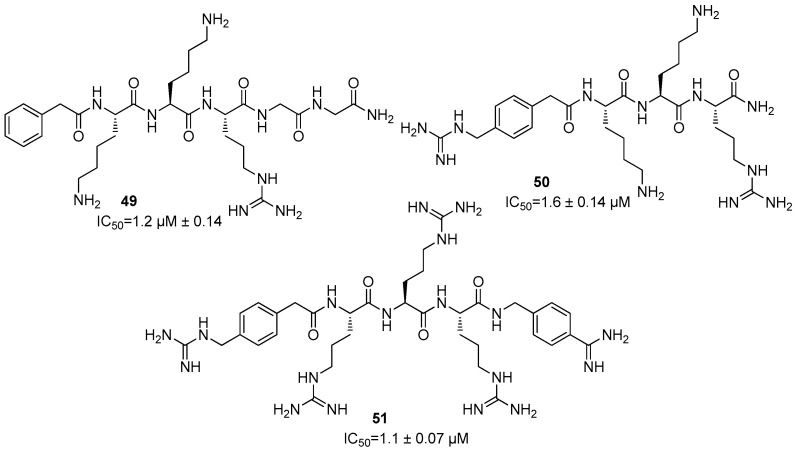
Inhibitors against NS2B/NS3 protease from ZIKV.

In addition, peptidomimetic inhibitors **49**–**51** were structurally characterized from three to five residues that mainly differ for the group linked to the *N*-terminal amino acid, showing against ZIKV NS2B/NS3 protease IC_50_ values of 1.2 ± 0.14, 1.6 ± 0.14, and 1.1 ± 0.07 µM, respectively (Figure 22) [22]. 

**Figure 23:**
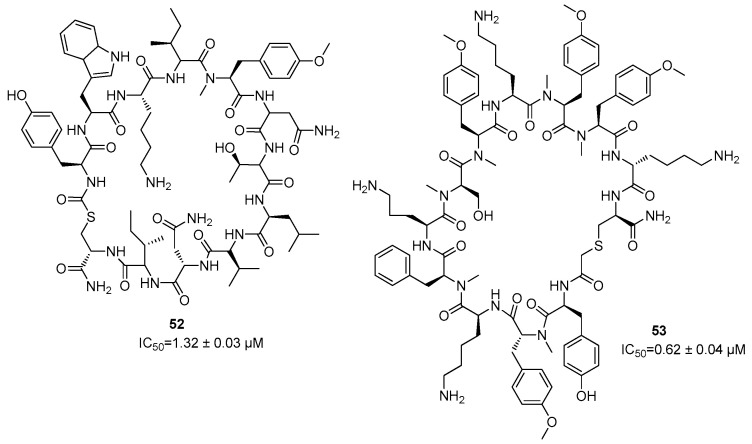
Macrocyclic inhibitors **52** and **53** of NS2B/NS3 protease from ZIKV.

Starting from small macrocyclic peptides (>2 kDa), further cyclic inhibitors against ZIKV were developed with the aim to improve their inhibitory potency. The most active compounds (**52**, **53**) are able to covalently bind to the catalytic serine of the active site, and they showed IC_50_ values of 1.32 ± 0.03 µM and 0.62 ± 0.04 µM, respectively (Figure 23) [76,77,78].

## 8. Pharmacokinetic Properties, Antiviral Activity, and Cytotoxicity Evaluation of DENV and ZIKV Inhibitors

Peptides are among the ideal candidates to be used as an alternative therapeutic option to conventional drugs, in different therapeutic fields. As can be seen in this perspective article, most of the orthosteric inhibitors show a peptide structure, and even if endowed of a potent antiviral activity, some limitations, regarding pharmacodynamics and pharmacokinetics properties, restricted their applicability in the pharmaceutical market [79].

For example, the low bioavailability of peptides, limited by their degradation and low epithelial absorption, is the highest difficulty in the therapeutic application of peptides. Another important problem is the degradation of peptides by gastric juices and by peptidases present in the gastro-intestinal tract. Even if the peptides escape this degradation route, another relevant problem to solve is the crossing of the intestine’s epithelial barrier, since peptides have to overcome the mucosal layer (composed of glycocalyx, glycoproteins, mucopolysaccharides, enzymes, water, and electrolytes), the brush border membrane with microvilli, and the efflux pumps, like P-glycoprotein, that is able to pump after absorption the peptide back into the gastro-intestinal lumen. In addition, after absorption, peptides can face some other problems like the first-pass effect, thus reducing their bioavailability in the systemic circulation. To overcome these problems, the peptides can be administered subcutaneously, intravenously or intramuscularly. However, parenteral administration does not guarantee that the peptides are delivered to their site of action, since the endogenous proteases may lead in any case to proteolytic degradation [79].

The antiviral activity of the orthosteric inhibitor **37** was specifically evaluated against Huh-7 cells infected by DENV-2, thus showing an EC_50_ = 3.42 µM. At the same time, the cytotoxicity of compound **37** was evaluated, obtaining a CC_50_ > 100 µM and a selectivity index SI > 25. 

The discreet passive membrane permeability of compound **37** was further assessed using the parallel artificial membrane permeability assay (PAMPA); in addition, considering the peptidic nature of this inhibitor and its metabolic clearance, the metabolic stability of **37** was assessed using liver microsomes from rats, obtaining a half-life of 175 min. Thus, considering its membrane permeability and its metabolic stability, compound **37** showed improved properties in DENV cell-based assays with respect to its parent molecules [61]. 

The permeability of compound **32** was also assessed with PAMPA, thus obtaining a Pe = 1.90 × 10^−6^ cm/s, that is a good starting point for peptide-based molecules, indicating the ability of this compound to pass the biological membranes and enter cells [58].

## 9. Conclusions and Perspectives

Proteases are generally ubiquitous in all life forms and are essential to many organisms, such as viruses, bacteria, and protozoa, since they regulate a number of cellular processes by catalyzing the enzymatic degradation of proteins. 

Within the drug discovery process for the treatment of DENV and ZIKV infections, the protease NS2B/NS3 is considered the primary target for the development of novel antiviral drugs.

The NS2B/NS3 is a trypsin-like serine protease that has a dual function both in the viral replication process and in the innate immunity; the NS3 protease acts through a catalytic triad composed by His51/Asp75/Ser135 residues located in a cleft between the *β*-barrels. The NS2B protein acts as a cofactor of the NS3 protease, undergoing a conformational change during the binding to NS3, necessary to activate the protease. 

As showed in this perspective, the aim of our work was to discuss the main features of the most active NS2B/NS3 inhibitors. So, among the allosteric inhibitors within the proline-based compounds, it has been highlighted that the compound **7** represents the most active inhibitor against ZIKV protease, with an IC_50_ value of 0.32 µM, while compound **2** showed the best inhibition against DENV2 protease, with an IC_50_ value of 4.2 µM. In Vero cells, compound **7** was proven to suppress the viral replication, thus decreasing the viral genome copy at a concentration of 3 µM. 

Within the pyrazine-based inhibitors, compound **23** showed against ZIKV, DENV2, and DENV3 proteases with submicromolar IC_50_s (IC_50_ = 0.20 µM, 0.59 µM, and 0.52 µM, respectively). 

Concerning the orthosteric inhibitors, the most promising DENV2 inhibitor is thiazolidinylcarbonyl-Arg-Lys-(OCH2C6H6(4-CF3)-Phg-NH_2_ (**36**), which showed an IC_50_ value of 18 nM, whereas compound **48** was the most active ZIKA NS2B/NS3 protease inhibitor, with an IC_50_ value of 280 nM. 

A central issue for the development of potent antiviral drugs is the enhancement of the antiviral activity at the cellular level, in such a way as to overpass the discrepancy that often may occur between the enzymatic activity and the efficacy against appropriate cell lines infected by ZIKV and DENV. 

To enhance the cell permeability, prodrug approaches, like the design of suitable carrier-linked prodrugs, have been proposed as a tool for increasing the intracellular uptake of inhibitors. Finally, novel technologies such as the incorporation of drugs into liposomes or nanocarriers might offer new opportunities to develop potent NS2B/NS3 inhibitors into efficacious antiviral agents. 

## Data Availability

Not applicable.
